# Cesarean Scar Pregnancy With Surgical and Conservative Managements: A Case Report

**DOI:** 10.7759/cureus.61311

**Published:** 2024-05-29

**Authors:** Banafsheh B Shoai, Lauren Gibbs, Regina Leonis

**Affiliations:** 1 Obstetrics and Gynecology, Morehouse School of Medicine, Atlanta, USA

**Keywords:** cesarean hysterectomy, methotrexate, cesarean scar, ectopic pregnancy, cesarean scar pregnancy

## Abstract

A cesarean scar pregnancy (CSP) is a rare form of ectopic pregnancy. Proper diagnosis and management of CSP are incredibly important secondary to the risk of uterine rupture and life-threatening hemorrhage. Various medical and surgical management have been described previously. This report looks at two cases of CSP diagnosed at an urban hospital in Atlanta, Georgia. The first woman was 30 years old with a history of five prior CS. She was referred from an abortion clinic for CSP at 6 weeks 2 days gestation. She did not desire future fertility and opted for a hysterectomy. The second woman was 38 years old with a history of three prior CS presenting with vaginal bleeding and abdominal pain and found to have a CSP with a gestation sac measuring 5 weeks 1 day. Given the patient’s desires for future fertility, she was treated with a two-dose regimen of systemic intramuscular methotrexate (MTX) at 1 mg/kg with successful resolution of CSP and subsequent intrauterine pregnancy. Due to the high risk of uterine rupture and hemorrhage with CSP, it is important to have a high index of suspicion for diagnosis. Due to the rarity of CSP, and thus difficulty creating quality prospective trials, there is no consensus on the best management yet. Although conservative treatment carries high failure risk, shared decision-making incorporating future fertility desires should be considered when determining management of CSP, and when surgical management is considered a minimally invasive approach should be the standard of care in surgical management.

## Introduction

Ectopic pregnancies account for about 1-2% of total pregnancies [1, 2]. A cesarean scar pregnancy is a rare form of ectopic pregnancy defined as the implantation of the pregnancy into the myometrial defect of the previous uterine scar [3]. The incidence of cesarean scar pregnancy (CSP) ranges from 1/800 to 1/2500 pregnancies [2, 4] and appears to be increasing with increasing rates of CS in the United States [1, 5]. Proper diagnosis and management of CSP are highly important to avoid uterine rupture and life-threatening hemorrhage. Transvaginal ultrasound (TVUS) has become the primary method of diagnosis of CSP with a sensitivity of 84% to 86.4% [6, 7], and magnetic resonance imaging (MRI) can be useful for the detection of placental implantation or bladder wall invasion [1, 8].

Two types of CSP have been noted. Type I or endogenic type is diagnosed by a gestational sac that is progressing toward the uterine cavity from its implantation point in the scar with a myometrial thickness of >3 mm [4, 6]. This type of CSP has the potential to reach viability but has a high risk of hemorrhage and placenta accreta spectrum complicating the pregnancy [3]. Type II CSP is partially located in the scar with a myometrial thickness of <3 mm [4]. Type III or exogenic type is located completely within the area of the scar with a myometrial thickness of <3 mm [4]. Type III grows toward the abdominal cavity/uterine serosa and is associated with deeper implantation and early uterine rupture [4, 6]. Vaginal bleeding and abdominal pain are common presenting symptoms of CSP [1, 2]. However, up to 1/3rd of patients with CSP may be asymptomatic and are diagnosed when they present for routine prenatal ultrasound [6]. The Crossover Sign (COS) may also be noted on ultrasonography and can be an indication of the type of CSP. COS is calculated by drawing a straight line (in sagittal view) connecting the internal cervical os with the uterine fundus through the endometrium; the superior-inferior (S-I) diameter of the gestational sac is then measured [4]. In the case of CSP, the gestational sac is implanted within the previous scar and the crossover line measures the S-I diameter of the gestational sac in relation to the endometrial line. In COS-1 at least two-thirds of the S-I diameter of the gestational sac is above the endometrial line, while in COS-2 less than two-thirds of the S-I diameter of the gestational sac is above the endometrial line [4]. The COS-1 group presented with higher rates of uterine rupture as well as placenta accreta spectrum when compared to the COS-2 groups [4].

Many methods have been described to manage CSP including expectant management [4], systemic or intra-gestational sac MTX, hysteroscopic suction, dilation and curettage (D&C), uterine artery embolization (UAE), hysteroscopy or D&C, hysteroscopic (MyoSure) resection [2], wedge resection and uterine repair, hysterectomy, and different combinations of the above [2-4, 7, 9-14]. In a systematic review of 32 studies including 3380 CSP patients, 583 patients were able to successfully conceive following fertility-sparing management of their initial CSP [5]. The probability of recurrent ectopic pregnancy in general was 16.6% for these patients while the probability of a recurrent CSP (RCSP) was 15.3% [5]. Women with a history of conservatively managed CSP still have a high pregnancy rate, but the risk of RCSP and spontaneous miscarriage is also increased [5]. However, it is not yet possible to clarify or compare different treatments due to the heterogeneity of treatments given, potential selection bias arising from reports of successful management in literature, and paucity of quality prospective and long-term data.

## Case presentation

Case 1

**Figure 1:**
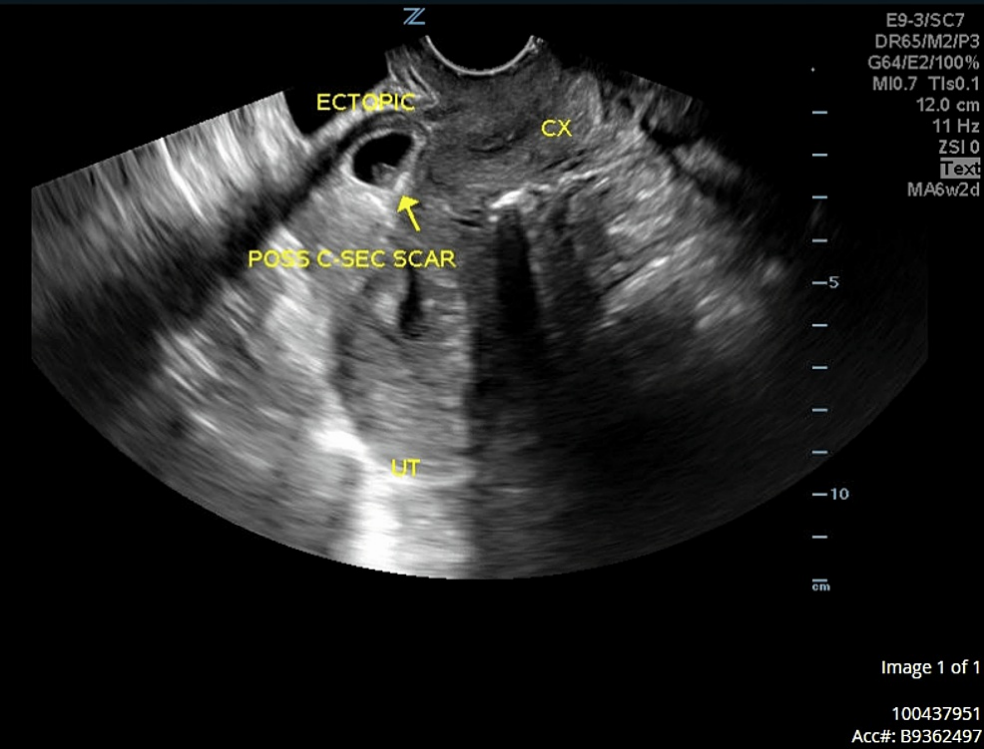
Transvaginal ultrasound (TVUS) (sagittal view) showing fetal pole at the level of the previous CS scar Cx: cervix; UT: uterus

A 30-year-old gravida 7 para 5 woman with five prior CS was referred to our institute from an abortion clinic for suspected CSP. She was asymptomatic at the time of presentation and reported that she did not desire future fertility. Her vital signs and physical exam were unremarkable. Hemoglobin (Hgb) was noted to be 12.6 gm/dL and b-human chorionic gonadotropin (hCG) was 26,072 mIU/mL. A fetal pole measuring 6 weeks 2 days and fetal cardiac activity (FCA) in the lower uterine segment at the level of the previous CS scar was noted on the TVUS (Figure 1).

After counseling, the patient was presented with management options available including intramuscular methotrexate, laparoscopic removal of CSP, and definitive management with robotic-assisted total hysterectomy. After a discussion of the risks and benefits of each option, she was concerned with the risk of failure with medical management given elevated b-hCG and FCA on ultrasound. Importantly, she did not desire future fertility. Therefore, she opted to proceed with a robotic-assisted total hysterectomy and bilateral salpingectomy. The uterus was extracted intactly and bivalved in the operating room. The intact gestational sac was found at the defect of the lower uterine segment covered only by very thin fibrotic tissue and no overlying myometrium. The pathology exam confirmed the gross findings and was noted to show "products of conception identified consisting of chorionic villi and fetal fragments" within a "hypersecretory endometrium." She was discharged on post-operative day 1. She did not follow up for postop clinic visit and eight weeks later presented with vaginal cellulitis after resuming sexual activities, which was successfully treated outpatient.

Case 2

**Figure 2:**
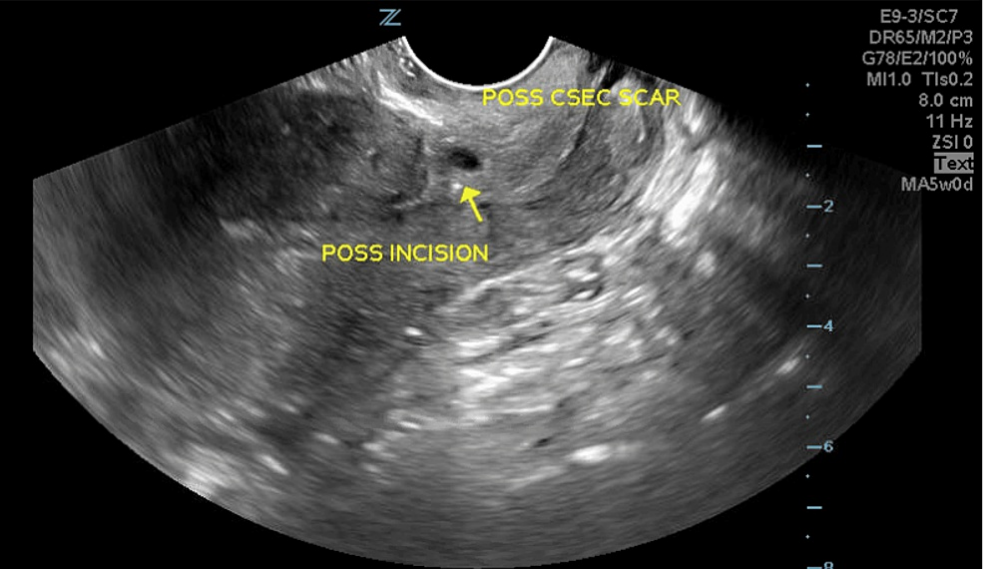
Transvaginal ultrasound (TVUS) showing gestational sac at the level of previous CS scar in the lower uterine segment

A 38-year-old Gravida 4 Para 3 woman with three prior CS presented to the obstetrics emergency room with complaints of vaginal bleeding and a dull aching pain in her lower abdomen starting four days ago. She reported a history of tubal ligation and subsequent reversal three years ago for desired future fertility. Vital signs and physical exam were unremarkable. Hgb was found to be 11.1 gm/dL and b-hCG 284 mIU/mL. TVUS showed an empty uterus and a gestational sac at the CS scar at the lower uterine segment with a mean sac diameter of 7 mm, estimating the gestational age to be 5 weeks 1 day (Figure 2). A possible yolk sac was noted without fetal pole.

The risks and benefits of both surgical and medical management were discussed with the patient for a CSP at this early gestational age. The patient highly desired future fertility and declined surgical intervention. With shared decision-making, she opted for two doses of systemic intramuscular MTX therapy dosed at 1 mg/kg on days 1 and 4, which were well tolerated without any symptoms or alterations in organ functions assessed by blood work. The b-hCG levels are listed below (Table 1).

**Table 1:** b-hCG level corresponding to day s/p MTX treatment

Day	b-hCG level (mIU/mL)
1	284
4	92
7	22
14	6

She was lost to follow-up until she presented back to triage four months later with an intrauterine pregnancy at 5w5d and progressed to have a full-term pregnancy delivered via repeat C-section.

## Discussion

With the increase in the rate of CS in the United States, there has also been an associated increase in the rate of CSP [1, 15, 16]. While both patients in this report did not experience CSP until after three or more CS, current data indicate that while the rate of CSP is increasing, the number of CS does not seem to be an independent risk factor for CSP as many cases occur after just one CS [17]. In a small study looking at risk factors for CSP, 85% (36/42) of women experienced CSP after only one prior CS [18]. Thus, CSP should be strongly suspected and ruled out when patients with even one prior CS present with vaginal bleeding or for their first ultrasounds. Failure to diagnose could result in significant maternal morbidity and mortality secondary to the risk of uterine rupture and hemorrhage. In Silva et al., a total of 492 cases of CSP were reviewed with 194 gestational outcomes described: 20.1% of cases resulted in miscarriage and 52.6% had a hysterectomy [4]. In the review by Morlando et al., the rate of subsequent intrauterine pregnancy was 83.4%, however, those with a history of CSP had a 15% to 34% chance of recurrent CSP with reports of some women experiencing up to five recurrent CSPs [16]. In the systematic review by Cali et al., 17 studies of patients with CSP that were managed expectantly were reviewed, and it was noted that in those with FCA, only 13% of patients experienced an uncomplicated miscarriage while 20% required medical intervention [15]. In those without FCA, 69.1% of women had an uncomplicated miscarriage. Forty women progressed to third-trimester pregnancy and 39.2% of those women had severe bleeding at the time of delivery [15]. A systematic review looking at managements of CSP published between January 1978 and April 2014, showed the following success rates for systemic MTX alone (8.7%), UAE (18.3%), hysteroscopy (39.1%), D&C (61.6%), and hysterotomy (92.1%) [9]. The subsequent hysterectomy rates for the above methods were 3.6%, 1.1%, 0%, 7.3%, and 1.7%, respectively [9]. A variety of dosages of MTX, such as 50 mg/m^2^ [4, 5], 1 mg/kg one-time systemic dose [7], various intra-gestational sac dosages, multi-dose regimens alternating with folinic acid [3, 10] have been reported. Administration of MTX was found to be ideal for those who presented before eight weeks gestation, with b-hCG concentration of <1200 mIU/mL and absent FCA [14]. Often different MTX regimens were used sequentially if there was not an appropriate drop in b-hCG after the first dose. Many studies reported small sample sizes due to the rarity of CSP, and therefore, the optimal regimen is still difficult to define.

## Conclusions

Currently, many methods have been described in the literature for the management of CSP including expectant management, systemic or intra-gestational sac MTX, hysteroscopic suction, D&C, UAE, hysteroscopy with D&C, hysteroscopic (MyoSure) resection, wedge resection and uterine repair, hysterectomy and combinations of the above. More evidence is needed to refine optimal management strategies of CSP due to highly heterogeneous treatment strategies and small sample sizes in prospective trials: multi-site prospective trials, reports of both successful and complicated outcomes, and subsequent pregnancy outcomes following different managements need to be encouraged. When deciding on management, shared decision-making should incorporate the patient's desire for future fertility in considering uterine-sparing options, however, they should be counseled for risk of current CSP, placenta accreta spectrum, uterine rupture, preterm birth, and miscarriage. Women undergoing CS should be counseled on the risks of future pregnancies, including the risk of CSP, importance of early prenatal care, and timely presentation for suspicious symptoms. Women who have completed childbearing should be counseled on reliable contraception, including bilateral salpingectomy during the CS or surgical management of CSP. When a surgical approach is considered, whether uterus-sparing or hysterectomy, a minimally invasive approach should be standard whenever possible.
